# Advancing pathogen genomics in resource-limited settings

**DOI:** 10.1016/j.xgen.2023.100443

**Published:** 2023-11-17

**Authors:** Paul Michael Pronyk, Ruklanthi de Alwis, Rebecca Rockett, Kerri Basile, Yann Felix Boucher, Vincent Pang, October Sessions, Marya Getchell, Tanya Golubchik, Connie Lam, Raymond Lin, Tze-Minn Mak, Ben Marais, Rick Twee-Hee Ong, Hannah Eleanor Clapham, Linfa Wang, Yorin Cahyorini, Francisco Gerardo M. Polotan, Yuni Rukminiati, Eby Sim, Carl Suster, Gavin J.D. Smith, Vitali Sintchenko

**Affiliations:** 1 Centre for Outbreak Preparedness, Duke-NUS Medical School, Singapore 169857, Singapore; 2 Emerging Infectious Diseases Programme, Duke-NUS Medical School, Singapore 169857, Singapore; 3 Sydney Infectious Diseases Institute, The University of Sydney, Camperdown, NSW 2006, Australia; 4 Centre for Infectious Diseases and Microbiology – Public Health, Westmead Hospital, Westmead, NSW 2145, Australia; 5 Centre for Infectious Diseases and Microbiology Laboratory Services, NSW Health Pathology – Institute of Clinical Pathology and Medical Research, Westmead, NSW 2145, Australia; 6 Saw Swee Hock School of Public Health, National University of Singapore and National University Health System, Singapore 117549, Singapore; 7 Infectious Diseases Translational Research Programme, Department of Microbiology and Immunology, Yong Loo Lin School of Medicine, National University of Singapore and National University Health System, Singapore 117549, Singapore; 8 Singapore Centre for Environmental Life Sciences Engineering, National University of Singapore, Singapore 117549, Singapore; 9 Nanyang Technological University, Singapore 639798, Singapore; 10 Big Data Institute, Nuffield Department of Medicine, University of Oxford, Oxford OX3 7LF, UK; 11 National Public Health Laboratory, National Centre for Infectious Diseases, Singapore 308442, Singapore; 12 Bioinformatics Institute, Agency for Science, Technology and Research, Singapore 138671, Singapore; 13 Programme for Research in Epidemic Preparedness and Response (PREPARE), Ministry of Health, Singapore 169854, Singapore; 14 Center for Health Resilience and Resource Policy, Ministry of Health, Jakarta 12950, Indonesia; 15 Molecular Biology Laboratory, Research Institute for Tropical Medicine, Muntinlupa 1781, Metro Manila, Philippines

**Keywords:** genomics, pathogen genomics, surveillance, whole-genome sequencing, emerging infectious diseases, resource-limited settings

## Abstract

Genomic sequencing has emerged as a powerful tool to enhance early pathogen detection and characterization with implications for public health and clinical decision making. Although widely available in developed countries, the application of pathogen genomics among low-resource, high-disease burden settings remains at an early stage. In these contexts, tailored approaches for integrating pathogen genomics within infectious disease control programs will be essential to optimize cost efficiency and public health impact. We propose a framework for embedding pathogen genomics within national surveillance plans across a spectrum of surveillance and laboratory capacities. We adopt a public health approach to genomics and examine its application to high-priority diseases relevant in resource-limited settings. For each grouping, we assess the value proposition for genomics to inform public health and clinical decision-making, alongside its contribution toward research and development of novel diagnostics, therapeutics, and vaccines.

## Graphical abstract

**Figure:**
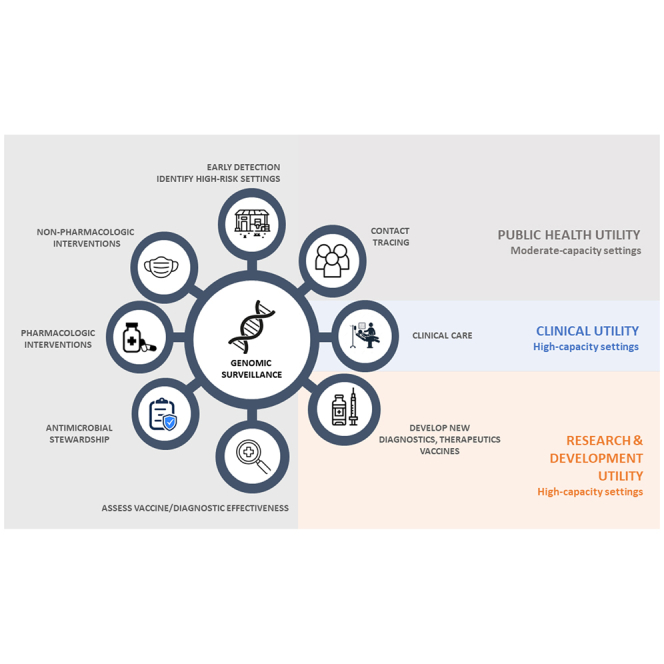

## Teaser

Pronyk et al. profile an approach to genomic surveillance for high-priority pathogens in resource-limited settings. National planning should be informed by local disease burdens, existing surveillance and laboratory systems, and the utility for genomics in each context. The aim is to design cost-efficient, multi-pathogen approaches that optimize public health impact.

## Introduction

Recent advances in pathogen genomics have transformed our ability to better detect and respond to infectious diseases.^1^ During the COVID-19 pandemic, genomic data associated with clinical and epidemiological information allowed public health officials to conduct real-time assessments of viral transmission and the potential impact of variants, which informed critical public health measures and supported evaluation of their efficacy. In addition to strengthening infectious disease surveillance, pathogen genomic information was also used to inform the rapid development, application, and evaluation of novel diagnostics, therapeutics, and vaccines.^2^

While genomic sequencing has been deployed extensively as a surveillance tool in high-income settings, major disparities exist in its application among low- and middle-income countries (LMICs).^3^^,^^4^ Prior to SARS-CoV-2, sequencing was largely absent from LMICs or deployed primarily as a research tool. Many low-resource settings still experience challenges implementing pathogen genomics including a lack of skilled personnel, inadequate laboratory infrastructure, and limited capacity to utilize genomic data in public health response.^3^^,^^5^ As genomic sequencing remains relatively expensive compared to more traditional laboratory testing methods, cost-benefit assessments are crucial in deciding where and how to implement pathogen genomics within existing surveillance and laboratory systems.

Despite these challenges, many LMICs have been exploring how to pivot genomic sequencing capacity built or expanded during COVID-19 toward priority endemic pathogens to enhance their infectious disease surveillance and control programs. To advance this agenda, the World Health Organization (WHO) announced its Global Surveillance Strategy for Pathogens with Pandemic and Epidemic Potential (2022–2023)^6^ and established the International Pathogen Surveillance Network.^7^ These global coordination mechanisms are supported by parallel efforts at the regional level, including the Africa and Asia Pathogen Genomics Initiatives (Africa and Asia PGI).^8^^,^^9^ These regional initiatives seek to accelerate pathogen genomics in parts of the world where the communicable disease burden is highest. Recent assessments highlight that while many LMICs in Asia and Africa have in-country capacity for pathogen genomics, few had comprehensive national plans that integrate pathogen genomics into wider surveillance efforts.^10^ Furthermore, while a multi-pathogen “public health approach” to sequencing has been identified as a crucial strategy, particularly where staffing and resources are more limited, global and regional guidance in this area is lacking.^3^^,^^8^

To address these gaps in genomics implementation planning, we summarize how recent advances in pathogen genomics can accelerate early disease detection and propose a framework to inform its introduction in low-resource settings. We further explore the value proposition for genomic surveillance across a range of high-priority disease groupings prioritized by LMICs in Asia, including tuberculosis, antibiotic-resistant bacteria, respiratory pathogens, zoonotic pathogens with high spillover risk, and arthropod-borne viruses, as well as the detection of emerging pathogens and the implementation of environmental surveillance as an early detection platform.^10^ The aim of this evolving effort is to develop a pragmatic evidence-based approach for policymakers on the integration of genomic surveillance within national disease control programs to optimize cost efficiency and public health impact.

### Advances in sequencing technologies

The last two decades have witnessed the rapid advancement of sequencing technologies.^11^ Second-generation technologies, also known as short-read sequencing (i.e., the sequencing of short DNA fragments), are currently the most abundantly used technology for pathogen sequencing (Table 1).^12^ The recent development of third-generation technologies, termed long-read sequencing due to the ability to sequence much longer nucleic acid fragments, provides additional benefits to infectious disease surveillance, such as easier genomic sequencing of novel pathogens. This includes innovations such as the commonly used Oxford Nanopore Technologies devices that are both highly portable and require limited additional laboratory infrastructure and are therefore readily deployable in field settings.

**Table 1:** Strengths and limitations of technologies and approaches commonly used for pathogen genomic sequencing

Pathogen genomic sequencing			
Technology or approach	Description	Strengths	Limitations
**Technology**			

Second generation	massively parallel sequencing with read lengths of 75–300 bp; most commonly used platforms are from Illumina, Ion Torrent (Thermo Fisher), and MGI	higher per-read-sequencing accuracy, therefore better at identification of single-nucleotide variants (SNPs), small insertions, and deletions; short reads allow for greater read depth for a given GB of output; lower cost per GB at high throughput	repetitive/homologous regions and structural variants pose difficulty in sequence assembly; amplification bias
Third generation	sequencing of native DNA; capable of reaching read lengths up to and greater than 10 kb; long-read technologies are provided by Oxford Nanopore Technologies and PacBio	longer read lengths allow greater genome coverage; advantageous for *de novo* assembly and sequencing of novel pathogens better for sequencing repetitive regions and structural variants (such as large insertions, deletions, duplications, or translocations) advantageous for genomic resolution of plasmids (which often carry antibiotic-resistant genes)	lower per-read accuracy; more stringent requirements for input quality and quantity

**Approach**^a^			

Amplicon-based	a targeted sequencing approach involving PCR amplification of genes or genetic material from the pathogen of interest, followed by sequencing	usually the cheapest sequencing approach and often easiest to implement and integrate with existing laboratory processes; because of PCR amplification, low input sample material required and higher likelihood of obtaining sufficient depth; relatively straightforward sequence assembly and bioinformatic analysis; less data storage and processing infrastructure required	prior knowledge of infecting pathogen required; possible PCR bias; in situations where circulating strain differs in the primer-binding regions, this may lead to gaps in resulting genome
Probe-based	utilizes synthetic probes to capture genes or genomes of interested pathogens; captured genomic material is then sequenced	able to capture a range of pathogens need less prior knowledge of exact infecting pathogen; greater uniformity of coverage; relatively straightforward bioinformatic analysis	longer and more laborious workflows; can be more expensive than amplicon-based sequencing due to cost of probe sets
Metagenomics	a non-targeted approach that sequences all genetic material (i.e., all pathogens and host nucleic acid) in the sample; metagenomics workflows can involve some prior treatment steps to reduce host material	allows for discovery of highly divergent strains or novel and rare pathogens; relatively easy and less time-consuming laboratory workflow	complex and heavy bioinformatic analysis; high data storage and processing infrastructure needed; most costly sequencing approach per sample; privacy concerns around sequenced host human genomics data

a Strengths and limitations for amplicon-based, probe-based, and metagenomics approaches described here are for culture-free applications.

Culture-free pathogen genomic sequencing can include three main approaches, i.e., (1) amplicon-based (targeted) amplification and sequencing of predefined pathogens, (2) probe-based (enrichment) for capturing and sequencing a range of known pathogens, and (3) metagenomics for untargeted sequencing. Amplicon-based sequencing is the most straightforward, cheapest, and most widely used approach for SARS-CoV-2 and other pathogens.^13^ Recent advances in bioinformatic analysis pipelines have enabled the adoption of metagenomics, which can detect both known and novel pathogens.^14^ Both probe-based genomics and metagenomics allow us to cast a wider pathogen net and therefore are becoming increasingly important for pathogen detection in undiagnosed clinical cases, animals, and environmental surveillance. Further innovations in portable sequencing have improved the accessibility of these technologies in both low-resource and remote settings.^15^ A cost-effective and pragmatic approach would be to design national pathogen genomic strategies that utilize a mix of these three sequencing approaches.

### Framework for prioritizing pathogens for genomic surveillance

**Figure 1:**
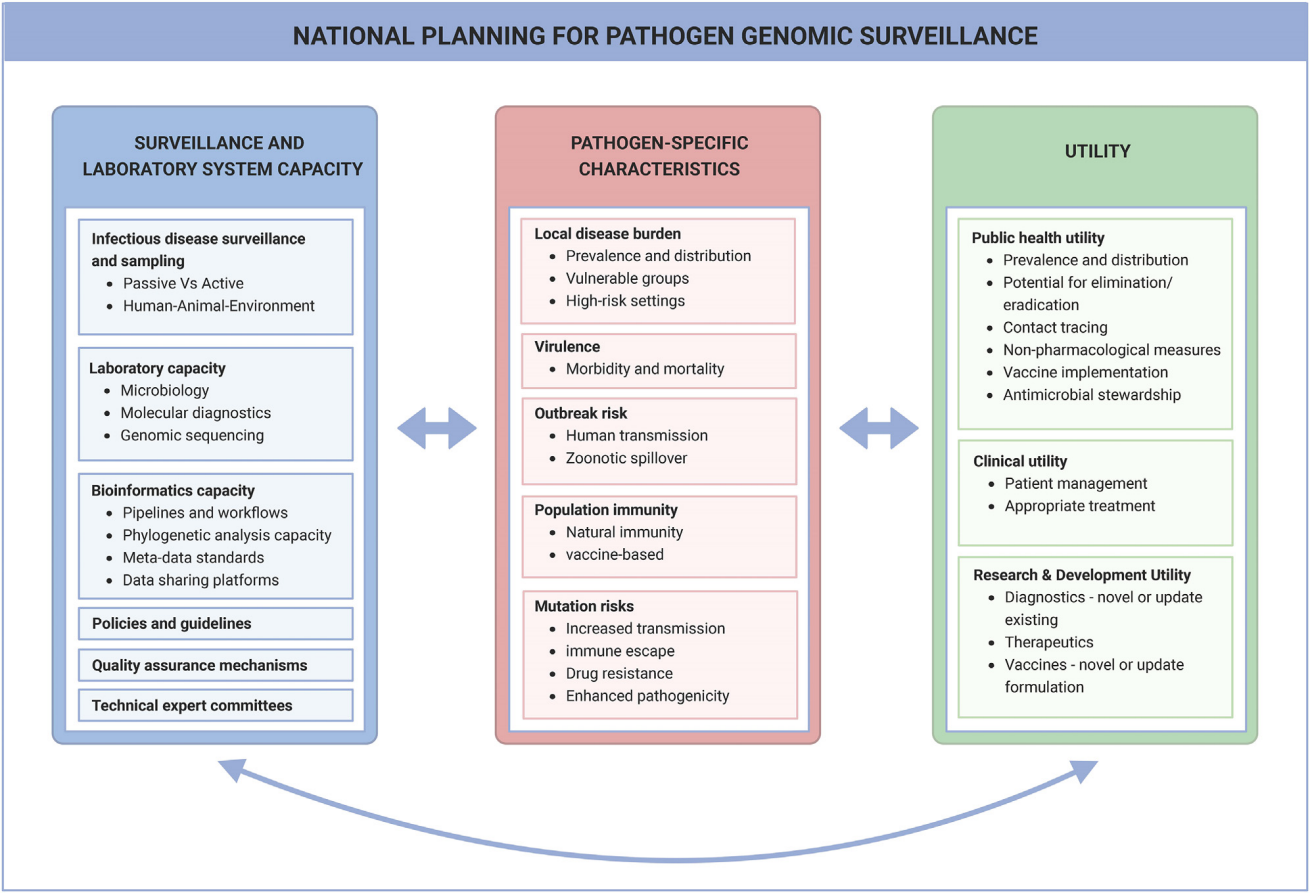
Conceptual framework for assessing the value proposition of pathogen genomic surveillance A framework was developed to help policymakers determine the utility of applying genomic surveillance for various pathogens. The framework considers three inter-related components: (1) existing surveillance and laboratory system capacity, (2) pathogen-specific characteristics, and (3) public health, clinical, and research and development utility.

In the context of the Asia Pathogen Genomics Initiative, we developed a framework to inform the utility of applying genomic surveillance to various pathogens and groupings across a spectrum of surveillance and laboratory system capacities. This framework has three interrelated components that should be weighed together to inform national planning. These include (1) existing surveillance and laboratory system capacity, (2) pathogen-specific characteristics, and (3) the potential to inform public health, clinical, or research and development (R&D) utility (Figure 1).

### Surveillance and laboratory system capacity

Our framework incorporates a pathogen genomics capacity assessment of national surveillance and laboratory systems, drawing upon an examination of existing tools, expert review, and country input. Detailed methodology and results from a 13-country assessment using this approach are profiled elsewhere.^10^^,^^16^ Key parameters included the robustness and coverage of existing infectious disease surveillance programs for sample collection, case detection, and/or appropriate referral; available laboratory capacity including turnaround times between sample collection and sequencing; bioinformatics and computing infrastructure for local data analysis; the existence of appropriate policies and guidelines; laboratory accreditation and external quality assurance programs; and the availability of technical expertise to advise on the most relevant genomic information to provide to public health officials and policymakers.

**Figure 2:**
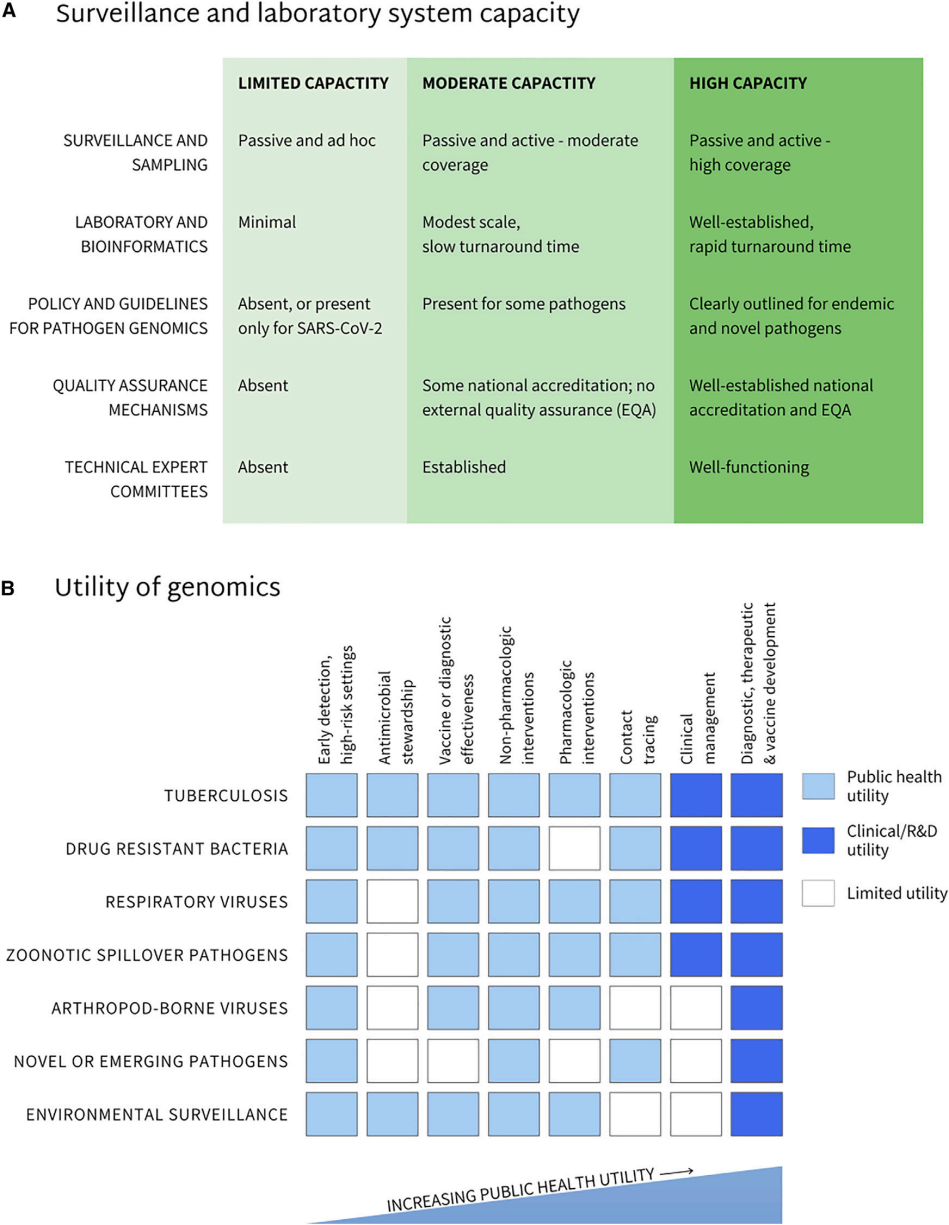
Utility of genomic pathogen surveillance by pathogen grouping based on level of surveillance and laboratory system capacity (A) Existing surveillance and laboratory system capacity determines the overall utility of applying genomic surveillance to various pathogens. The capacity to implement pathogen genomics has been categorized into three tiers: limited, moderate, and high capacity. (B) Areas of public health (light-blue shaded boxes), clinical, and research and development (R&D) (dark-blue shaded boxes) where the utility of pathogen genomics has informed decision-making in moderate and high-capacity settings are shown. These utilities have been applied to a number of high-priority pathogen groupings including (1) tuberculosis, (2) drug-resistant bacteria, (3) respiratory viruses, (4) zoonotic viruses with high spillover risk, (5) arboviruses, (6) novel or emerging pathogens, and (7) environmental surveillance (as a platform).

Country profiles from this assessment highlight wide variations in pathogen genomics capacity.^17^ The major limitation affecting the ability of pathogen genomics to inform timely public health responses was lengthy delays between sample collection and reporting of sequencing results. This effort informed the development of a simplified three-tiered typology (i.e., low, medium, and high capacity) that allows countries to self-assess national or sub-national readiness for surveillance and laboratory systems to implement pathogen genomics (Figure 2).

### Pathogen-specific characteristics

Within each country and context, pathogen prioritization for genomic surveillance should also be informed by the epidemiological, biological, and clinical characteristics of pathogens and associated diseases.^18^^,^^19^ Epidemiological parameters include measures of local disease burden, such as national prevalence, geographic distribution, and risk-group characterization; population levels of natural and vaccine-derived immunity; and pathogen-specific risks of local transmission, such as human-to-human transmission or zoonotic spillover. Important biological and clinical characteristics may include the rate of genetic mutations and the consequences of new variants with respect to pathogenicity, transmission, immune escape, and drug resistance (DR).

### Utility

The inclusion of a specific pathogen within national genomic surveillance guidelines should be linked to priority actions that require such genomic information. These considerations are especially vital in resource-constrained regions. Our framework outlines three areas where pathogen genomic surveillance has been shown adding value to decision-making, including (1) infectious disease control measures (public health utility); (2) patient care and treatment (clinical utility); and (3) the development of outbreak mitigation or prevention tools, such as diagnostics, therapeutics, or vaccines (R&D utility).^20^

### Pathogen and capacity-specific applications of genomic surveillance

We adopt a public health approach to profile the utility of integrating pathogen genomics within national infectious disease control programs. First this entailed a focus on high-priority pathogen groupings that contribute to the burden of disease or outbreak risk and have been identified as country priorities, in this case in Asia, but with broader relevance elsewhere. Second is the ability to leverage existing or evolving surveillance systems for pathogen detection. Third is the need to emphasize cost efficiency. Finally, we focused on groupings where timely identification can lead to clinical or public health action and improvements in outcomes. The pathogens or groupings included in this review are (1) tuberculosis (TB), (2) drug-resistant bacteria of public health concern, (3) respiratory pathogens, (4) zoonotic viruses with high spillover risk, (5) arthropod-borne viruses, (6) novel or emerging pathogens, and (7) environmental surveillance as an early detection platform (Figure 2).

Our review and collective experience suggest that the immediate value of introducing pathogen genomics is likely to be low in contexts where basic surveillance and laboratory capacity is severely limited. In these settings, investments in advancing basic laboratory testing and disease response capacity should be prioritized alongside integrating genomics into pathogen surveillance. We therefore focus our discussion mainly on moderate- and high-capacity contexts recognizing that a range of capacities exist both within and between countries.

### Tuberculosis

As the leading cause of global morbidity and mortality, interrupting *Mycobacterium tuberculosis* (*M. tuberculosis*) transmission and responding to the threats posed by DR are a global priority.^21^^,^^22^ A number of genome sequencing techniques have been applied to TB. Sequencing can be performed after *M. tuberculosis* isolation on selective media or directly from clinical specimens. Sequencing options include post-culture whole-genome sequencing (WGS), pre-amplification targeted sequencing of specific parts of the TB genome that predict DR, and TB metagenomics, which sequences all host and microbial nucleic acid in a clinical sample (refer to Table 1 for sequencing technologies and approaches).^23^ Genome sequences of *M. tuberculosis* strains circulating in high-burden countries remain underrepresented in global databases. However, reductions in sequencing costs, standardization of laboratory and bioinformatics protocols,^24^ alongside the release of the WHO catalog of DR-conferring mutations^25^ have increased the public health and clinical utility of *M. tuberculosis* sequencing.^26^ Given the infrastructure and bioinformatics requirement of sequencing, its implementation in low-capacity settings could be challenging and may not be a high-priority investment to improve TB control.

In moderate-capacity settings where *M. tuberculosis* transmission is usually endemic (incidence >50 per 100,000 population), the aims of genomic surveillance are primarily to (1) strengthen TB control efforts, (2) inform empiric programmatic treatment, and (3) identify high-transmission settings where additional public health interventions might be undertaken (i.e., active TB case finding or expanded use of TB preventive therapy). Where frontline molecular diagnostics for TB have been adopted including the use of tools such as GeneXpert, sequencing of specimens identified as rifampicin resistant or multi-drug resistant (RR/MDR), particularly in relapsed patients in whom resistance was not identified previously, is important to inform better targeted empiric treatment regimens, for instance by identifying genetic mutations associated with resistance to traditional first- and second-line as well as novel anti-TB drugs. Rapid DR detection by frontline molecular diagnostics facilitates the identification of priority specimens for culture and WGS. Sequencing plays an important role in comprehensive DR surveillance and for monitoring emerging and novel DR mutations^27^ by identifying mutations occurring in relapsed cases in whom DR was not detected by frontline molecular diagnostics.^28^ Taken together, this information offers great value to monitor the appropriateness of empiric DR-TB treatment regimens and the risk of misdiagnosing DR-TB cases using frontline molecular tests and for identifying potential DR-TB transmission hotspots, particularly in healthcare settings. With the expectation of more effective TB vaccines in the near future, genomic monitoring will also help to assess vaccine effectiveness, as well as potential strain selection and immune escape.^29^

In higher-capacity settings where incidence rates are generally below 50 cases per 100,000 population, genomic surveillance is mainly used to track TB elimination progress with the aim of “zero local TB transmission” and to guide optimal personalized care in drug-resistant cases. Monitoring TB transmission is important to ensure the safety of the local population despite a high percentage of imported cases arising in recent migrants^30^ and to define enhanced programmatic benchmarks for TB control.^31^ Routine sequencing is particularly useful to identify likely transmission pathways,^28^^,^^32^ differentiate relapse from re-infection,^33^^,^^34^ and to recognize laboratory contamination events.^35^ For DR, routine sequencing may replace phenotypic drug susceptibility testing of first-line drugs with associated cost savings, while also providing a comprehensive overview of potential resistance against second-line and novel anti-TB drugs. Sequencing of drug-resistant strains provides valuable guidance to clinicians regarding the most appropriate personalized treatment regimen to consider if done early.,^36^^,^^37^ Given the urgency required for clinical decision-making, this is the setting where culture-independent targeted next-generation sequencing (tNGS) is likely to be most applicable, given the current high costs associated with these tests. However, wider tNGS implementation for DR surveillance and better tailored treatment in high TB incidence settings is keenly anticipated.^38^

### Drug-resistant bacteria

Antimicrobial resistance (AMR) is a major global public health threat largely driven by the excessive and inappropriate use of antimicrobials.^39^ While higher rates of AMR have been documented in LMICs relative to high-income countries (despite lower levels of antibiotic consumption),^40^ the current understanding of AMR dynamics in low-resource settings is limited.^41^

The main source of global data on AMR prevalence is derived from phenotypic antimicrobial susceptibility testing (AST) from bacteriologically confirmed infections. While AST provides direct evidence of resistance, it does not elucidate the mechanism of resistance.^42^ Innovations in sequencing allow pathogen genomes to be rapidly assessed for established phenotypic and genetic markers of AMR. When linked to clinical, AST, and epidemiological data, genomic information can assist in understanding transmission dynamics and uncover novel genetic elements that reduce antibiotic susceptibility.

As LMICs are disproportionately affected by AMR, establishing strong surveillance networks with the capacity for targeted WGS is a high priority. A prerequisite for introducing WGS for AMR in moderate-capacity settings is the existence of an AMR surveillance program where microbiology laboratories have the capacity to conduct phenotypic AST. Understanding the background characteristics of AMR within a population is a necessary starting point to inform whether newly detected AMR strains are pre-existing or imported. This can be achieved through retrospective sequencing of stored isolates, with a focus on WHO priority pathogens and locally important species. This process can inform local prioritization for prospective periodic sampling based on relevant public health concerns, associated research questions, and potential links to local agriculture and food export. Prospective passive surveillance of susceptible and resistant isolates should also be accompanied by targeted surveillance of outbreaks and unusual isolates.^16^

Genomic surveillance for AMR in moderate-capacity settings offers numerous public health and economic benefits. First, it informs decisions regarding antimicrobial stewardship, where the use of narrow-spectrum drugs may reduce AMR emergence. Second, WGS for AMR can help enhance the capacity of microbiology laboratories in contributing to public health surveillance. Third, the detection of AMR in health facility isolates can support early identification of nosocomial transmission or transmission threats and can support the introduction of public health measures such as intensified infection prevention and control (IPC). Fourth, in many LMICs, sequence findings have guided vaccine development and implementation, such as the inclusion of the pneumococcal conjugate vaccine into the childhood vaccination schedule.^43^ Fifth, conducting WGS on bacterial pathogens that may be linked to local agricultural production can help establish potential transmission sources, enabling the introduction of mitigation measures to ensure local food safety and retain the confidence of food export markets.^16^^,^^44^ Sixth, the establishment of national genomic databases for bacterial pathogens can contribute to global efforts to better understand transmission patterns and AMR evolution dynamics.^39^ Finally, while genomics would generally be deployed in tandem with phenotypic/culture-based surveillance, AMR sequencing also holds promise in helping to better understand mutations conferring resistance with the aim of overcoming bottlenecks in settings where conventional microbiology systems are not sufficiently robust.^16^^,^^45^

In high-capacity settings, the overuse of broad-spectrum agents has resulted in bacterial pathogens that respond to few, if any, available antimicrobials.^46^ Alongside informing decisions regarding antimicrobial stewardship and vaccine introduction for bacterial pathogens,^47^ real-time sequencing can enhance contact tracing efforts to help pinpoint transmission routes. For healthcare-associated infections, sequencing can assist in determining whether outbreaks have emerged *de novo* or have been introduced from outside sources, which carries implications for enhanced IPC or public health measures. Genomic data can also be used to identify accompanying mutations and/or gene acquisition that may be linked to shifts in transmission or virulence.^48^ Finally, understanding the AMR marker repertoire of specific pathogens may influence clinical care decisions where individual patients may not be responding to treatment, alongside informing the development of customized molecular diagnostics and therapeutics.

### Respiratory pathogens

Acute respiratory infections are a major cause of morbidity and mortality with disease burdens in LMICs estimated to be 10–50 times greater than in high-income countries.^49^ Nearly 80% of these infections are caused by common viral pathogens including influenza, respiratory syncytial viruses, parainfluenza viruses, adenoviruses, and human rhinoviruses. Furthermore, recent outbreaks of viral respiratory pathogens have occurred due to SARS-CoV-2, human and avian influenza, and Middle East Respiratory Syndrome coronavirus (MERS). The COVID-19 pandemic was among the first instances where rapid genome sequencing of a respiratory pathogen profoundly assisted with disease control efforts.

While SARS-CoV-2 was an important illustration of how rapid genome sequencing of a respiratory pathogen can assist with disease control efforts, genetic surveillance for influenza and respiratory syncytial virus has been increasing integrated into existing disease surveillance strategies in the last decade.^50^^,^^51^ Notably, the unprecedented global adoption of rapid genomic surveillance for disease control during the COVID-19 pandemic was attributed to leveraging on WHO Global Influenza Surveillance and Response System (GISRS) partnership with the GISAID initiative.^52^^,^^53^ Many national influenza centers within GISRS have since expanded their capacity to perform genomic sequencing during the pandemic, and specimens collected for influenza and SARS-CoV-2 surveillance can potentially be exploited to characterize the burden of other respiratory pathogens.^54^ However, culture-independent genomic sequencing of respiratory specimens is complex given the large amount of host genetic material, the wide range of potential respiratory pathogens, the frequency of respiratory virus co-infection, and the presence of commensal organisms that may not be contributing to disease.

Combining molecular diagnostics with sequencing is essential for cost-effective surveillance of pathogenic respiratory viruses. In most instances, respiratory samples will first be screened using conventional techniques, such as rapid antigen tests or multiplex PCR panels, which have shorter turnaround times and simpler technical requirements.^55^^,^^56^ Samples with positive detection of a respiratory virus may then be subjected to amplicon-based targeted genome sequencing. For specimens that test negative by conventional virus-testing panels, more complex and expensive metagenomics can be performed to sequence and identify the viral etiology of undiagnosed respiratory disease.^9^

In moderate-capacity settings, countries should prioritize the application of conventional microbiology, antigen tests, and molecular diagnostics as the most cost-effective approach for detecting widely circulating respiratory bacterial and viral pathogens. Given the recent expansion of in-country sequencing capacity, national public health institutions and academic/research bodies are now well positioned to sequence influenza and other respiratory viruses. The value proposition is largely public health focused, where sequencing can provide further insights into outbreak detection and variant analysis and support the detection of unknown pathogens in patients with severe respiratory illness who test negative using conventional diagnostic methods.

Even within high-capacity settings with well-functioning surveillance and laboratory systems, the use of sequencing for systematic assessment of respiratory pathogens remains at an early stage. Recent research programs such as the Respiratory Virus and Microbiome Initiative in the United Kingdom aim to establish routine sequencing-based surveillance methods to better understand transmission dynamics of circulating viruses.^9^ Currently amplicon-based methods are most commonly used to sequence respiratory pathogens. These methods need to be specifically designed and validated for each individual respiratory virus. Viral enrichment using multi-pathogen probe-based hybridization potentially offers significant advantages to laboratory workflows. These probe panels may be designed to simultaneously sequence a broad range of respiratory pathogens in a single laboratory workflow. The enrichment probes also require less sequence homology with the targeted virus than amplification primers, limiting the requirement for laboratories to redesign primers due to mutations developing in primer regions. Although routine genomics capacity for respiratory viruses is still in its infancy, expanding and supporting wider sequencing capacity are crucial for early outbreak detection for respiratory pathogens and to support the development of diagnostics, therapeutics, and vaccines. In these contexts, the combination of conventional microbiology, molecular diagnostics, and innovations in genomic sequencing can also be used to inform clinical care decisions.^57^

### Zoonotic pathogens with high spillover risk

Most human infectious diseases, including pathogens with high outbreak risk, originate from animals.^58^ Recent decades have witnessed repeated human outbreaks of viruses originating in wild or domestic animal reservoirs including HIV, swine and avian influenza, MERS, Ebola, Nipah virus, SARS-CoV, SARS-CoV-2, and others. Shifts in population density and migration, climate change, species loss, and patterns of human-animal interaction suggest the risk of zoonotic spillover will increase in the future.^59^ Pathogen genomics is a powerful tool to detect zoonotic spillover events, understand transmission dynamics, inform public health measures, and assist in the development of novel response tools.^60^

Low-resource countries, particularly those in Africa and Asia, are the areas of the world at highest risk of zoonotic disease and spillover events.^59^ Many of these are likely undetected and under-reported, and hence, active and sentinel surveillance for zoonotic pathogens using a One Health framework is crucial.^61^ In these settings, the deployment of molecular diagnostics that target pathogens of local importance can be combined with sequencing to enable early detection of zoonotic spillover. As levels of viremia in many spillover events remain low, serology can be used as a complimentary tool to screen for potential exposure among high-risk populations. Guidance exists for risk stratification at human-animal interfaces, such as wet markets,^62^ and to reduce potential outbreak threats through disease surveillance among domestic, farmed, and wild animals.^63^

The main utility of sentinel surveillance in moderate-capacity settings remains early detection of disease threats and the introduction of public health measures. These measures include informing outbreak investigation and contact tracing, assessing the potential for human-to-human transmission, introducing non-pharmacological interventions (physical distancing, handwashing, respiratory hygiene, regulatory changes, waste management), and the use of human or animal vaccination where appropriate. Furthermore, such measures ensure the confidence and security of local domestic and wildlife markets, which have important economic benefits.

In high-capacity settings, zoonotic outbreak risk has declined over time through the introduction of public health measures and guidelines for animal care in the agricultural sector. Nonetheless, zoonotic spillover of bacterial pathogens such as salmonella, brucella, and leptospirosis; protozoa such as toxoplasmosis; and viral pathogens such as SARS-CoV-2, influenza, West Nile virus, and rabies remains a serious public health concern.^64^ In these contexts, genomic sequencing has been used extensively for animal health, to help pinpoint the animal source and dynamics of transmission, and to inform the design of outbreak mitigation and control tools.

### Arthropod-borne viruses

Arboviruses include several viruses of public health significance such as dengue, zika, Japanese encephalitis, yellow fever, and chikungunya viruses, with the largest burden in LMICs. With almost half the world’s population at risk from an arboviral infection, this group of viruses represents one of the largest public health threats to those living in tropical and sub-tropical regions.^65^ Due to globalization and climate change, arthropod vectors of transmission (such as mosquitoes and ticks) have gradually expanded their geographical habitat, increasing the number of cases and outbreaks over recent decades.^66^

The diagnosis of arboviruses such as dengue is mainly based on clinical symptoms, serology, and molecular diagnostics such as PCR. As antivirals are generally ineffective against arboviruses, treatment is usually supportive. Therefore, arboviral genomics currently play little to no role in the clinical care of patients with arboviral disease.

Most arboviruses are RNA viruses. While RNA viruses have relatively high potential mutation rates due to poor proofreading mechanisms, arboviruses tend to have much lower observed evolutionary rates. For example, studies with dengue virus have shown that the lower evolutionary rate is a result of its transmission cycle between human and mosquito hosts, which exerts multiple genetic bottlenecks.^67^ These issues are complex, however, given that substitutions take place in the context of two host systems. Nonetheless, these restricted mutation rates mean that low-resource settings can utilize a less exhaustive sampling strategy for genomic surveillance of arboviruses, where changes in genotype or serotype can anticipate increased hospitalizations.

Despite the bottlenecking phenomenon, arboviruses such as dengue do contain high genetic diversity leading to strains being classified into serotypes, genotypes, and clades.^68^ Sequencing can be done either from clinical samples from patients or from mosquitoes. Though in some cases viremia may be low upon presentation to healthcare settings, recent advances in sample preparation prior to sequencing have enabled the recovery of a high percentage of full viral genomes.^69^ Sequences can also be sourced from mosquitoes (with pooled mosquito samples). However the yield in mosquitoes is often low. In moderate-capacity settings, genomic characterization of acute febrile cases remains important for early detection of new strains, identification of high-risk populations, predicting potential outbreaks, and identifying possible sources of importation.^68^ In the case of dengue, detection of new genotypes will often alert national public health authorities to initiate a range of non-pharmacological interventions, such as mosquito control through release of *Wolbachia* mosquitoes, fogging, and mosquito traps alongside wider community awareness.^70^ Additionally, arbovirus genomic surveillance can inform vaccine immunization campaigns. As the first licensed dengue vaccine (Dengvaxia) led to greater risk of severe disease with dengue virus serotype 2 and given that another dengue vaccine has been recently licensed (TAK-003),^71^ it is imperative that endemic countries are able to track their locally circulating dengue strains using genomics-informed surveillance. The extent to which genomic diversity will impact the success of the vaccines or Wolbachia is unclear. Therefore, it is important to closely monitor circulating strains of dengue within populations as preventative measures, such as vaccines and Wolbachia, are rolled out.^72^

In high-capacity settings, passive surveillance and sampling of acute febrile cases should be accompanied by active surveillance and sampling of household contacts to capture asymptomatic infections.^73^ In addition to human surveillance, arboviruses are also frequently sampled, sequenced, and identified in mosquitoes, providing a complimentary surveillance strategy.^74^ This is also useful to track global transmission patterns including timing and extent of exportation and importation. Historically, the application of genomics to arboviruses has mostly focused on sequencing surface viral glycoproteins, which has been the major target of diagnostics and vaccine design. Recent studies have shown, however, that regions outside of the structural genes may play an important role in pathogenicity and epidemic potential.^75^ Additional studies involving WGS of arboviruses should be conducted to build a more complete map of the genomic regions that contribute to outbreak dynamics.

### Novel pathogen detection

In the context of outbreaks from a potentially unknown pathogen, genomic sequencing plays an essential early detection role given that traditional molecular diagnostics and serological assays may not exist. After identification of an index case, epidemiological investigations assess for clustering and the potential source of an infection. Microbiological and molecular diagnostic testing are conducted to rule out common pathogens. In cases where molecular testing is inconclusive, sequencing can be deployed using approaches such as targeted enrichment panels. In conjunction, metagenomics and/or meta-transcriptomics can be performed to detect unknown pathogens. Once a pathogen is genomically detected and confirmed, genomic data can be used to develop PCR-based molecular diagnostics that can be deployed at scale at a much lower cost.

Given recent advances and cost reductions in sequencing, even moderate-capacity settings have the capacity to detect unknown pathogens. The main challenge will be the identification of suspected cases in clinical settings and the collection, handling, and transport of high-quality samples to sequencing laboratories. To be of public health value, sequencing must be embedded and integrated within well-functioning national surveillance systems. Establishing sentinel surveillance programs in high-risk settings such as intensive care units or areas of high zoonotic spillover risk will likely provide the greatest utility while broader national capacity is being developed. Expansion of capacity will require support for continuous clinical surveillance, sampling, and/or referral of specimens to a national or regional network of reference laboratories with adequate biosafety levels and increased capacity for bioinformatics and data sharing.

In high-capacity settings, a global agenda to respond to “Disease X” has recently been defined with the aim of making safe and effective vaccines, therapeutics, and diagnostics available within 100 days of identifying a new disease threat.^76^ Genomics has enabled the rapid development of novel tools to respond to outbreak threats. Studying so-called prototype pathogens has been put forth as a critical element of preparedness.^77^ By developing vaccines on rapid-response platforms against exemplars of a given viral genus or family, researchers can address scientific challenges characteristic of that family in advance, providing an important head start on developing vaccines against related threats.

### Environmental surveillance

Pathogen surveillance through wastewater sampling has emerged as an important early warning tool to detect outbreak threats. During the COVID-19 pandemic, SARS-CoV-2 was tracked extensively using wastewater sampling across high- and low-resource countries, with detection of variants of concern 7 to 14 days ahead of clinical surveillance.^78^ The WHO has recently introduced guidance for environmental surveillance to augment routine SARS-CoV-2 surveillance systems.^79^ Wastewater surveillance has also been applied for a range of other pathogens, including bacterial AMR, typhoid, cholera, and human enteroviruses including polio.^80^ Finally, metagenomics on environmental samples has been identified as a new approach to identify and characterize unknown potential pathogens before they emerge.^81^

In moderate-capacity settings, environmental wastewater sampling can be utilized in a strategic low-cost manner. In situations of low endemicity and rare symptomatic case incidence, environmental surveillance may detect outbreak threats in advance of clinical surveillance. For polio, environmental sampling is particularly important as acute flaccid paralysis occurs in fewer than 1% of infections.^82^ Most LMICs employ a combination of viral culture and PCR followed by traditional (Sanger) sequencing methods to distinguish wild-type from vaccine-derived polio.^83^ However, this method is slow and leads to delays in sequencing. In some settings, polio genomic sequencing using next-generation techniques has been used to ensure a faster time between sampling and detection.^84^ In this instance, the value of sequencing would be for early detection of polio transmission in the population, to inform polio vaccine implementation strategies, and to support global eradication efforts.

Targeted sequencing has also been deployed in moderate-capacity settings to detect known viral pathogens and AMR bacteria using environmental surveillance. For diseases of high national and global importance, it is potentially cost effective to leverage existing polio wastewater sampling capacity to conduct targeted surveillance using either molecular diagnostics or targeted sequencing to identify known pathogens.^79^ The advantage of wastewater surveillance is that samples can be collected easily in areas where clinical AST may be limited and can detect resistant bacteria circulating in the community. The disadvantage is that the link between mutations and clinical resistance is unclear.^85^ In LMICs, wastewater-based epidemiology using pooled samples has been suggested as an inexpensive approach relative to mass clinical testing to estimate SARS-CoV-2 prevalence, to track clusters and trends in the general population, and to identify hotspots in large cities or areas.

Importantly, metagenomic approaches for sequencing of pathogens in wastewater samples face a number of technical challenges.^86^ It is comparatively expensive and can only detect low abundance organisms with sufficiently high sequencing depth, and even then, it is unlikely to be able to reconstruct genomes of bacteria with meaningful completeness or coverage. Furthermore, bioinformatics analysis of metagenomic sequencing of environmental samples remains complex.

Culture-enriched metagenomics represents an emerging strategy that is a compromise between metagenomics and WGS. For bacterial pathogens, this involves sequencing an entire selective enrichment culture instead of WGS of individual colonies or metagenomics of environmental samples. It also has the advantage of recovering low-abundance pathogens and yielding quality draft genomes. However, it is not quantitative and does not capture the whole pathogen community present in an environmental sample.^87^

In high-capacity systems, innovative applications of environmental surveillance have recently been introduced given the relative ease of sample collection and its potential to deepen understandings of pathogen transmission dynamics. Aircraft-based wastewater surveillance using targeted sequencing and metagenomics has been used to identify emerging viruses, trace their evolution, and map global spread with international flights.^88^ Surveillance is also done for aquatic environments beyond water-quality testing. Water bodies are considered good proxies for surrounding locales, as water drains microbes from animals, soils, and plants into lakes or reservoirs.^87^ Finally, innovations in active air-sampling have been deployed as a high-throughput surveillance tool to identify respiratory viruses and variants in congregate settings with the capacity to detect SARS-CoV-2 on a smaller timescale and closer proximity to the source in comparison to wastewater surveillance.^89^

### Future directions

Genomic surveillance has emerged as an important tool for early detection of novel and endemic pathogens in low-resource settings. This review emphasizes that the utility of pathogen genomics is likely to be greatest where surveillance and laboratory systems have been established with existing capacity to respond to genomic signals. In severely resource-constrained settings, capacity will likely have to be built across multiple areas before in-country pathogen genomics can feasibly be adopted and deliver the impact. Interim measures to out-source sample sequencing and data analysis to higher-capacity settings will likely be the only option. These should target high-priority pathogens such as influenza and diseases poised for eradication or elimination such as polio.

However, building upon recent gains during the COVID-19 pandemic, many LMICs have achieved sufficient surveillance and laboratory capacity where pathogen genomics can make an important contribution toward disease control efforts. Pathogen genomics can inform a wide range of public health measures to strengthen surveillance systems, guide antimicrobial stewardship, and guide decisions regarding the introduction of pharmacological and non-pharmacological interventions. The pathogen groupings where the public health applications of genomics are more well-established include TB, AMR, respiratory pathogens, and assessment of zoonotic spillover. Efforts to expand capacity for outbreak investigation and early detection of novel pathogens through environmental and One Health surveillance remain an urgent priority.

We reviewed the major categories or groupings of pathogens and diseases that contribute to the burden of disease and have been prioritized for pathogen genomics among LMICs in Asia but with broader relevance elsewhere. These pathogen groupings also reflect commonalities in disease epidemiology, laboratory diagnosis, and public health surveillance and response. They are not intended to be exhaustive but rather illustrative of the potential value and trade-offs of integrating pathogen surveillance within national planning. There is unlikely to be a one-size-fits-all approach given local, national, and regional variations in surveillance and laboratory system capacity, disease epidemiology and response capacity. Nonetheless, the experience gained in pathogen genomics targeting endemic disease will be invaluable in detecting early signals of novel pathogens.

While the cost of sequencing has come down substantially in recent years, genomic surveillance remains relatively expensive. Depending on the choice of sequencing platform, initial investments to establish sequencing and bioinformatics capacity are estimated to be US$100,000–$700,000.^90^ Recent estimates of the cost per sequence ranging from US$20–$200, with poor countries paying up to 10-fold more than high-income countries.^91^ Sequencing platforms rely upon high-throughput to keep costs down. Price inequalities result from inefficiencies in system use, small volume purchases, limited numbers of suppliers and distributors, and delays in customs clearance, which affect the ability to optimize the use of time-sensitive biological reagents. To facilitate planning in the design of cost-efficient systems for multi-pathogen surveillance, countries will require technical support to optimize platform selection and in forecasting requirements for consumables with the aim of realizing cost reductions through batched procurement.

Countries remain heavily reliant on external partner support, which contributes to over half the costs of pathogen genomics in Asia and likely more elsewhere.^8^ Leveraging global financing mechanisms such as The Global Fund and The World Bank’s Pandemic Fund will be critical to provide additional resources to support immediate improvements in early detection capacity among LMICs.^92^ In an effort to respond to procurement challenges, genomics equipment and reagents have recently been listed in global supply catalogs, which creates opportunities for price reductions through aggregating demand and pooling procurement across countries.^93^

However, to enhance long-term sustainability, prioritized multi-pathogen surveillance planning will be essential. Countries require support in the cost-efficient use of pathogen genomics for endemic pathogens through effective surveillance planning, sampling, and system design. Recognizing that significant differences in capacity exist within resource-limited contexts, the positioning of genomic surveillance systems should optimize both cost efficiency and public health impact. In some instances, centralizing capacity with efficient models for sample collection and transport may be optimal; whereas in areas with more geographically localized disease risk, establishing decentralized capacity may be more appropriate. Furthermore, as capacity is often spread between national public health institutions, the private sector, and academic/research entities, decisions on how to integrate pathogen genomics into routine public health response as opposed to more focused research efforts remain an important consideration.

Technical issues should also be considered carefully in weighing decisions regarding system design. This includes how to deploy sequencing alongside more cost-effective diagnostic microbiology and molecular testing for screening of known pathogens. The availability of complementary approaches is particularly relevant for TB, AMR, and respiratory pathogen detection. The effective pooling of samples and multiplexing of detection assays may offer important cost savings though can result in longer turnaround times for delivering sequencing results. This hampers the ability of genomic sequencing to be used for timely contact tracing and clinical decision-making in areas where capacity is limited. Finally, the use of metagenomics to detect novel and unknown pathogens remains a more expensive proposition with fewer bioinformatic workflows and higher complexity of result interpretation. While metagenomics is an appealing tool for detecting novel and unculturable pathogens, it should be deployed cautiously in settings where national genomics capacity is still evolving.

In settings with more well-established systems, pathogen genomics carries wider utility and can further disease elimination efforts. Enhanced surveillance, laboratory, and bioinformatics capacity results in shorter turnaround times between sample collection, sequencing, and reporting, alongside the ability to more readily deploy metagenomics on clinical or environmental samples for novel pathogen detection. This allows for more timely outbreak investigation, source attribution, and the initiation of public health measures. Regarding clinical utility, pathogen genomic data can personalize therapeutic options for conditions such as drug-resistant TB and drug-resistant bacterial and respiratory disease and can improve patient outcomes and drug utilization as the result. Finally, locally generated genomic data can enhance national R&D capacity and standing through contributing to the design of novel diagnostics, therapeutics, and vaccines as well as sharing benefits of genomic data.

### Conclusions

Genomic sequencing as a public health surveillance enabler is a groundbreaking new tool, with the ability to overcome current inequalities in access to infectious disease diagnostics and control interventions. Prioritization of pathogens and conditions alongside cost-effective system design to optimize public health impact are particularly important in low-resource settings that are disproportionately affected by outbreak risk. The described framework should assist in the integration of pathogen genomic sequencing within national surveillance plans, establishing reporting mechanisms to inform real-time public health decisions, and effective cross-country genome data sharing. Finally, leveraging experience from the COVID-19 pandemic, coordinated global efforts to rapidly translate genomic data into novel pandemic response tools and enable access for all countries remain a central priority for regional and global health security.
